# Undernutrition and associated factors among lactating mothers in Chiro district, eastern Ethiopia: a community-based cross-sectional study

**DOI:** 10.3389/fgwh.2024.1440606

**Published:** 2024-09-12

**Authors:** Mesfin Fekadu, Gudina Egata, Bezatu Mengestie, Hassen Abdi Adem, Ahmedin Aliyi Usso

**Affiliations:** ^1^ West Hararghe Zone Health Office, Oromia Regional Health Bureau, Ministry of Health, Chiro, Ethiopia; ^2^ School of Public Health, College of Health and Medical Sciences, Haramaya University, Harar, Ethiopia; ^3^ Saint Paul’s Hospital Millennium Medical College, School of Public Health, Addis Ababa, Ethiopia; ^4^ School of Nursing and Midwifery, College of Health and Medical Sciences, Haramaya University, Harar, Ethiopia

**Keywords:** malnutrition, undernutrition, risk factors, lactating mothers, Ethiopia

## Abstract

**Background:**

Maternal undernutrition during lactation is a worldwide public health problem. It causes impaired cognitive ability, poor productivity, irreversible loss, and intergenerational malnutrition, which has harmful effects on the next generation. Overall, there is little information on undernutrition and risk factors among lactating mothers, especially in resource-poor settings, including Ethiopia. This study assessed undernutrition and associated factors among lactating mothers in rural Chiro district, eastern Ethiopia.

**Method:**

A community-based cross-sectional study was conducted among 629 lactating mothers in the Chiro district from July 2–30, 2019. Data were collected from participants using pretested, structured questionnaires and anthropometric measurements. Data were entered using EpiData version 3.1 and analyzed using SPSS version 27. Multivariable logistic regression analyses were used to identify factors associated with undernutrition.

**Results:**

Undernutrition among lactating mothers was 26.9% (95% CI: 23.2%, 30.2%). Female-headed household (AOR = 0.34, 95% CI:0.13, 0.94), medium (AOR = 0.58, 95% CI: 0.38, 0.95) and rich (AOR = 0.30, 95% CI: 0.18, 0.51) wealth quintiles, lack of dietary advice (AOR = 1.62, 95% CI: 1.10, 2.39), chewing khat (AOR = 1.82, 95% CI: 1.23, 2.70), low dietary diversity (AOR = 3.10, 95% CI: 1.82, 5.29), and household food insecurity (AOR = 3.67, 95% CI:1.47, 9.20) were factors significantly associated with undernutrition.

**Conclusions:**

Around one in every four lactating mothers in rural eastern Ethiopia had undernutrition. Poor wealth, lack of dietary feeding advice, substance use disorder, low minimum dietary diversity, and household food insecurity were factors significantly associated with the undernutrition of lactating mothers. Thus, focusing on implementing existing strategies/programs for effective nutritional interventions and poverty alleviation that enhance food security status would be essential to improving the nutritional status of lactating mothers and children.

## Introduction

Undernutrition is a worldwide public health problem accounting for losses of millions of lives every year. Globally, 795 million people are underweight, which increased by more than 167 million in the last decade (1). Maternal undernutrition is the underlying reason for 3.5 million deaths of total global diseases. Maternal undernutrition is more common in lower-income settings. Evidence shows that 5%–20% of African women have a low body mass index (BMI) (1–3).

Reproductive-age women (15–49 years) are the most nutritionally vulnerable due to high physiological demands during pregnancy and lactation (4, 5). In developing countries, women spend a larger proportion of their reproductive years on pregnancy and lactating. It is estimated that, on average, women in Africa and Asia spend 30%–48% of their reproductive lifetime in pregnancy or lactation (6). Moreover, frequent pregnancies followed by lactation raise the risk of maternal undernutrition and death. Malnourished women who have closely spaced pregnancies and heavy workloads during pregnancy and lactation replicate the intergenerational cycle of malnutrition (7–9).

Depending on its length and intensity, lactation can have various implications for a mother's nutritional status. Lactating mothers, particularly those living in low and middle-income countries (LMIC), are highly vulnerable to undernutrition (10). This is mainly due to increased physiological demand, the lactogenesis process, workload, poverty, inadequate food intake, poor nutritional quality of diets, and increased nutrient needs during lactation (5). The risk of malnutrition in women spans a lifecycle, and preventing maternal undernutrition during the first 1,000 critical days requires intervening at all stages of growth and development (7). The nutritional status of one generation of women affects the nutritional well-being of their children in childhood and adulthood, the intergenerational effect of undernutrition (7), and hence, adequate nutrition and healthcare for women are imperative for their health and that of their offspring.

Undernutrition among lactating women was assessed using the body mass index (BMI), the most frequently utilized standardized indicator of thinness, to assess progressive loss of body weight/energy in developing countries. BMI is defined as weight in kilograms divided by the square of the height in meters (kg/m^2^), using the cut-off points suggestive of underweight among women (BMI < 18.5 kg/m^2^) as recommended by the International Dietary Energy Consultative Group (4, 9).

Globally, around 2 billion people suffer from micronutrient deficiencies, and 793 million suffer from calorie deficiency each year (11). Out of 1.76 billion reproductive-age women worldwide, 528 million (30%) suffer from anemia, and 19 million (1.1%) are vitamin A deficient. One in every five deaths during pregnancy and childbirth was due to maternal undernutrition (8, 12).

Maternal undernutrition, chronic energy deficiency, and micronutrient deficiencies contribute to maternal anemia, which accounts for about 11% of the global burden of diseases (8). In Ethiopia, nearly a quarter (24%) and 22% of women aged 15–49 are anemic and underweight respectively (13). The burden of undernutrition among lactating women ranges from 9% to 49.2% in Asia (10, 12, 14), 1.25% to 30% in Africa (3, 15), and 15.8% to 59% in Ethiopia (16–18).

The major causes of undernutrition are inadequate dietary intake, preventable diseases/infections, educational status, family size, occupational status, and household food insecurity (18, 19). Moreover, across the globe, in resource-poor countries, low-quality diets, poverty, lack of access to adequate health services, and gender discrimination are the risks for a variety of micronutrient deficiencies (14, 20, 21).

Undernutrition has both short-term consequences (deaths, morbidity, and disability) and long-term consequences (stunting, impaired cognitive ability and growth development, poor productivity, poor reproductive performance, and rise in metabolic and cardiovascular diseases) (1, 5, 11). In addition, it has intergenerational consequences. In women, being grossly underweight can result in amenorrhea, infertility, and multiple complications during pregnancy and lactation. The consequences of maternal undernutrition affect not only the health and survival of women but also that of their children and also impact the nation's development (7).

The three main existing strategies for the prevention and control of hidden hunger and undernutrition across the world are short-term food supplementation, medium-term food fortification, and a long-term focus on dietary quality or diversification (22). Besides, there is no single food group that contains all the nutrients required for the healthy functioning and performance of the body; thus, there is a need for more food groups to be included in the daily diet (5). The Ethiopian Federal Ministry of Health (FMoH) has a goal to reduce maternal undernutrition through the implementation of different program interventions by health development armies, health extension programs, and launching a revised national nutrition program that emphasizes women of reproductive age, including lactating mothers and their children (23).

The proportion of women in reproductive groups suffering from malnutrition is significantly higher and rampant in rural areas, where poverty and hunger are the highest. The highest rates of women's undernutrition were reported at 48%, 42%, 39%, and 38% in Somali, Afar, Gambella, and Benishangul-Gumuz rural areas, respectively, while the lowest prevalence was in Shashamane (9.5%) (24), Addis Ababa (18%), and Harari (25%), urban areas (24–26). In addition, the previous studies were facility-based and focused on urban communities, which cannot represent the exact nutritional status of lactating mothers in rural communities (25–27). Despite the increased risks of women's malnutrition in rural areas, there is a scarcity of information on the magnitude of undernutrition and associated factors among lactating mothers in rural communities in eastern Ethiopia. Therefore, this study assessed the prevalence of undernutrition and associated factors among lactating mothers in the Chiro district in rural eastern Ethiopia.

## Materials and methods

### Study design and setting

A community-based cross-sectional study was conducted in the rural Chiro district in eastern Ethiopia from July 2–30, 2019. Rural Chiro district is found in West Hararghe Zone in Oromia Regional State, 323 kilometers east of Addis Ababa, the capital of Ethiopia. According to the Chiro district health office, the district has an estimated 232,672 total population (114,009 males and 118,662 females), 51,493 estimated women aged 15–49 residing, and 10,005 lactating mothers who had children less than 24 months in 39 rural kebeles in 2019. In 2019, there were seven health centers, 39 health posts, and 156 health professionals, with 62.3% antenatal care coverage and 55.2% postnatal coverage in the district. The district was one of the food-insecure malnutrition hotspot areas targeted in the productive safety net program for 29,669 households in all 39 kebeles. Common agricultural products were cereals (sorghum and maize) and some vegetables (cabbage, tomato, onions, and chills).

### Population

All lactating mothers in the rural Chiro district were the source population. All randomly selected lactating mothers in selected kebeles were the study population. An individual lactating mother who engaged in the study during the data collection period was the study unit. All lactating mothers whose breastfeeding children were aged 6–24 months and were permanent residents of selected kebele were included in the study. Those who were critically sick and mentally ill mothers who could not respond to interviews and those mothers who were physically deformed (that affects anthropometric measurement) were excluded from the study.

### Sample size determination and sampling

The sample size (*n* = 640) was calculated by Epi Info version 7.1 using a single-population proportion formula to assess the proportion of undernutrition among lactating mothers and a double-population proportions formula to identify factors associated with undernutrition among lactating mothers, and the larger one was taken as the minimum sample requirement. Accordingly, the largest sample size for this study was computed using single population proportion formula with the following assumptions: 25.4% proportion of lactating mothers undernutrition (25), confidence level of 95%, margin of error of 5%, design effect of 2% and 10% non-response, and thus, a minimum of 640 subjects were required.

**Figure 1:**
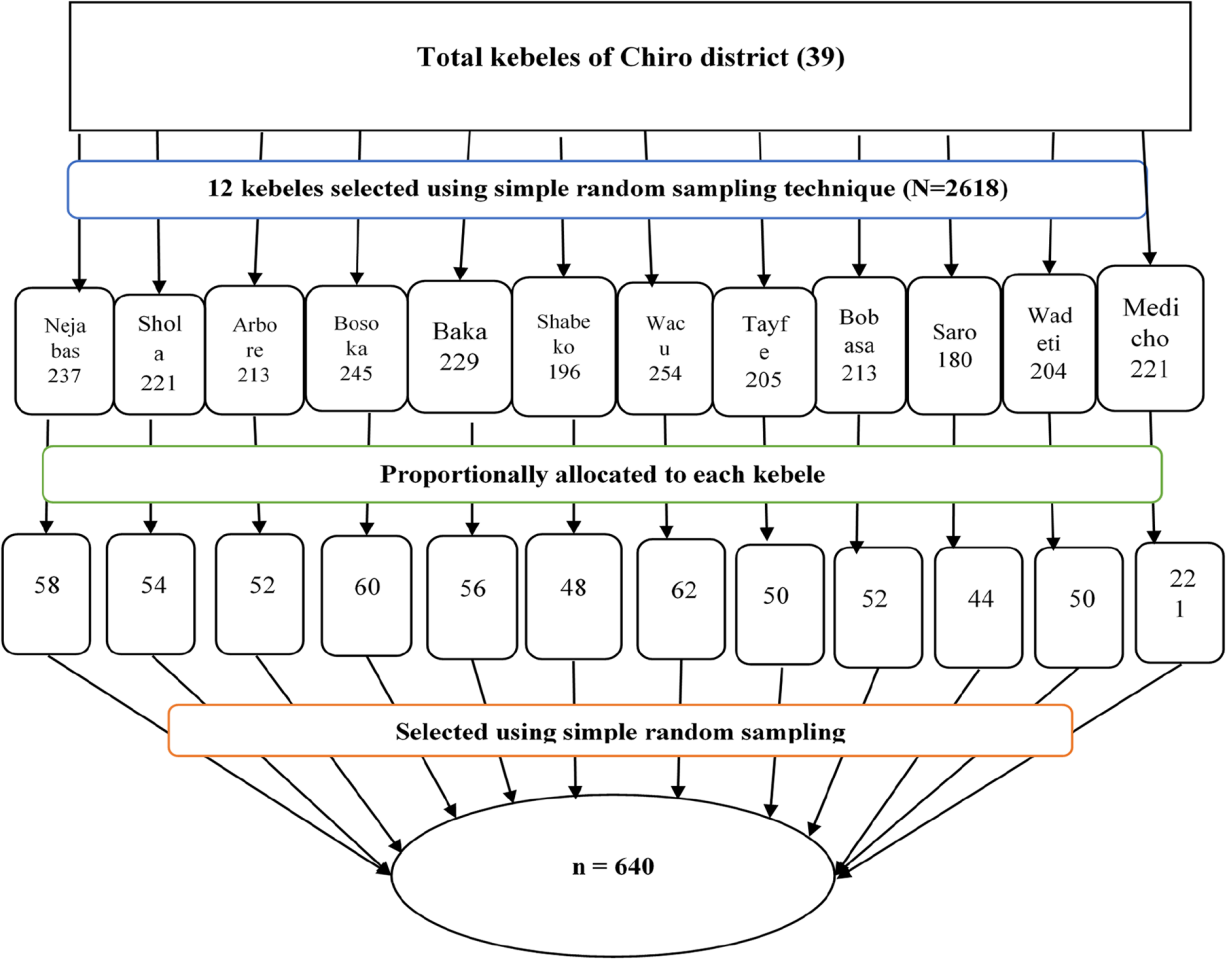
A flow diagram of sampling procedure.

We selected participants using a two-stage sampling method. Twelve out of 39 rural kebeles in the district were randomly selected. The house-to-house census was conducted in selected kebeles and eligible households with lactating mothers who had children 6–24 months recorded. Households with eligible mothers were assigned registration numbers to prepare the sampling frame, and 2,618 eligible lactating mothers were identified and recorded. The sample size was proportionally allocated to each randomly selected kebele based on the number of lactating mothers. A simple random sampling technique was used to select the study participants using household registration numbers as a sampling frame. Participants not present on at least three data collection trips were considered non-respondents. When there were two or more eligible mothers in one selected household, only one eligible mother was chosen by the lottery method (Figure 1).

### Data collection tools and procedure

The data was collected using pretested structured questionnaires adapted from relevant published literature (5, 20, 26, 28–31). The questionnaire contains information on socio-demographic characteristics, reproductive health factors, healthcare-related factors, household food security status, and dietary diversity status of mothers. The questionnaire prepared in English was translated into the local language (Afan Oromo) by two experts with a good command of both languages. Twelve data collectors were used to collect the data under the supervision of four supervisors. Anthropometric measurements (weight and height) and face-to-face interviews were used to collect the data from the lactating mothers. Lactating mothers’ weight and height were measured using a weighing scale with an attached height meter (Charger HM200P Stadiometer, Taiwan). The weighing scale was checked before and after each measurement using an object with a known weight to confirm accuracy. Before weighing, the mothers removed their shoes and wore light clothing during the anthropometric measurements. The mother requested to stand upright on a flat surface with their heels touching and their eyes looking straight ahead. The shoulders, buttocks, and shoulder blades should also be in contact with the vertical Studio-meter. The moving headpiece of the Studio-meter was applied to lower to rest flat on the top of the head, and the measurement read to the nearest 0.1 cm.

### Operational definitions

#### Body mass index (BMI)

BMI was calculated by dividing the mothers’ weight in kilograms by their height in meters squared (kg/m^2^). The lactating mothers were considered as undernutrition when their BMI was <18.5 kg/m^2^ and not otherwise (4, 9).

#### Minimum dietary diversity of women

The dietary diversity of lactating mothers was assessed using ten dichotomous (yes/no) food items (Cronbach's *ɑ* = 0.77) consumed in the last 24 hours. The composite index score was computed based on the responses to these ten items, which included food groups such as grains, white roots and tubers, plantains, pulses, nuts and seeds, dairy, meat and poultry, fish, eggs, dark green leafy vegetables, other vitamin A-rich fruits and vegetables, other vegetables, and other fruits. The mother's dietary diversity was “high” when consumed at least five food groups and “low” otherwise (5).

#### Household food insecurity

It was assessed using household food insecurity access scale (HFIAS) tools validated in Ethiopia (28). HFIAS has nine items (Cronbach's *ɑ* = 0.87) asking about household food access in the last four weeks and experiences about three domains of food security: uncertainty of food supply, insufficient quality of food, and insufficient food intake. The study participants were categorized as food secure if they responded “no” to nine questions and insecure if they responded “yes” to at least one question of HFIAS (31, 32).

**Wealth index:** was measured using a standard instrument containing 38 yes/no items arranged under three main domains (33). We observed high internal consistency among items (Cronbach's *α* = 0.81), and then we used principal component analysis using varimax rotation to determine composite wealth indexes and the wealth status of the participants.

### Data quality controls

The data quality was maintained using a standardized questionnaire adapted from a validated scale and relevant published literature. The questionnaire was first prepared in English and translated into the local language (Afan Oromo) by two experts with good command of both languages. The adapted questionnaire was pretested on 5% of the sample size to check its validity in separated non-selected kebele in the district. Twelve trained data collectors were used to collect the data under the supervision of four supervisors. The data collectors and supervisors were trained for two days on the objective of the study and data collection techniques. Supervisors and principal investigators strictly monitored data collection and validated the collected data.

### Data processing and analysis

After checking for completeness, the data were entered into EpiData version 3.1 and analyzed using SPSS version 27. The participants were characterized using descriptive statistics such as frequency, proportion, mean, median, range, and standard deviation. Wealth index score and wealth status of mothers were calculated using principal component analysis and the varimax rotation approach. Multicollinearity was checked between independent variables using the variance inflation factor. Bivariable logistic regression analysis was used to identify the association between each independent and dependent variable. Variables with *p*-value <0.25 in bivariable analysis are included in the multivariable model**.** Multivariable logistic regression analysis was used to identify factors associated with the undernutrition of lactating mothers using a backward stepwise likelihood ratio of model building. Hosmer and Lemeshow's goodness of fit test confirmed the model fitness with a *p*-value of 0.75. An adjusted odds ratio (AOR) with 95% CI was used to report association and significance declared at *p* < 0.05.

## Results

### Sociodemographic characteristics

A total of 629 (98.3%) lactating mothers were participated in the study. More than half (57.7%) of the participants were in the age group of 25–34 years, and the mean age ± SD of participants was 40 ± 10.34 years. Around three–fourths (76.2%) of participants have no formal education. Five hundred thirty-two (84.6%) were housewives, and 29.1% were poor. Regarding occupational status, more than five in every six (84.6%) of the mother's occupations were housewife (Table 1).

**Table 1:** Sociodemographic characteristics of lactating mothers in chiro district, eastern Ethiopia, 2019 (*n* = 629).

Characteristic	Frequency	Percent
Maternal age (in years)		
15–24	181	28.8
25–34	363	57.7
35–44	85	13.5
Religion		
Muslim	531	84.4
Orthodox	88	14.0
Protestant	10	1.6
Ethnicity		
Oromoo	541	86.0
Tigrai	10	16.0
Amhara	51	8.7
Other^a^	27	4.3
Maternal education status		
No formal education	479	76.2
Read and write	43	6.8
Primary education	101	16.6
Secondary education and above	6	1.0
Maternal occupation		
Housewife	532	84.6
Merchant	32	5.1
Daily laborer	60	9.5
Government employee	5	0.8
Marital status		
Married	599	95.2
Divorced/separated	17	2.7
Widowed/Single	13	2.0
Education status of HH head		
No formal education	357	56.8
Read and write	66	10.5
Primary education	127	20.2
Secondary education	54	8.5
Higher education	25	4.0
Husband occupation		
Farmer	562	89.3
Merchant	38	6.1
Daily laborer	29	4.6
Sex of household head		
Male	590	93.8
Female	39	6.2
Family size		
<5	194	30.8
≥5	435	69.2
Child age (in months)		
≤12	329	52.3
>12	300	47.7
Household wealth index		
Poor	183	29.1
Medium	202	32.0
Rich	244	38.9

^a^ Somale, Argoba.

### Health care and feeding related factors

Six out of ten (61.2%) lactating mothers had never attended antenatal care services, 61.7% had given birth at home; and 77.1% had at least four pregnancies, while 43.1% and 42.6% had a suboptimal birth interval and never used modern contraceptives, respectively. More than half (54.8%) of participants had received dietary advice. Around 56.6% had ≥3 meals per day, and 16.7% were feeding at least one additional food during the lactation period. Around three-forth, 476 (75.7%) lactating mothers consumed less than five varieties of food groups in the last 24 hours before the interview, and almost all (96.7%) participants consumed cereals (Table 2).

**Table 2:** Healthcare and feeding related factors of lactating mothers in chiro district, eastern Ethiopia, 2019 (*n* = 629).

Characteristic	Frequency	Percent (%)
Number of antenatal visits		
0	385	61.2
1–3	212	33.7
≥4	32	5.1
Place of delivery		
Health facility	241	38.3
Home	388	61.7
Birth interval		
First (NA)	71	11.3
<24 months	271	43.1
≥24 months	287	45.5
Gravida		
≥4	485	77.1
<4	144	22.9
Attend postnatal visit		
Yes	12	1.9
No	617	98.1
Modern contraceptives use		
Yes	361	57.4
No	286	42.6
Received nutrition advice		
Yes	345	54.8
No	284	45.8
Meal frequency per 24 h		
<3	273	43.4
≥3	356	56.6
Extra meal feeding		
Yes	105	16.7
No	524	83.3
Illness in the last 2 weeks		
No	570	90.6
Yes	59	9.4
Chewing khat use		
No	283	45.0
Yes	346	55.0
Minimum dietary diversity of women (MDDW)		
<5 food groups	476	75.7
≥5 food groups	153	24.3

MDDW, minimum dietary diversity of women; NA,  not applicable.

### Prevalence of undernutrition

Prevalence of undernutrition among lactating mothers was 26.9% (95% CI: 22.2%, 32.2%) with the mean (±SD) BMI of lactating mother 21(±2.5).

### Factors associated with undernutrition

In the bivariable analysis, nutritional advice, khat chewing, wealth status, minimum dietary diversity, household food security status, maternal age, sex head of household, gravidity and birth interval were significantly associated with undernutrition of lactating mothers. In multivariable analysis, chewing khat, sex of head household, wealth status, nutritional advice, minimum dietary diversity, and household food security status were the main determinants of undernutrition among lactating mothers. The model fitness was confirmed using the Hosmer and Lemeshow goodness of fit test with a *p*-value of 0.75.

The risks of undernutrition among lactating mothers were lower by 42% [AOR = 0.58(0.31, 0.95)] and by 70% [AOR = 0.30 (0.18, 51)] among those who belong to the medium and rich wealth quintiles compared to the poor. The odds of undernutrition of lactating mothers were 66% lower among mothers living in female-headed households [AOR = 0.34 (0.127, 0.94)] compared to their counterparts. The odds of undernutrition of lactating mothers were two times [AOR = 1.63 (1.11, 2.39)] higher among those who did not get dietary feeding advice compared to those who got it. The odds of undernutrition among lactating mothers were nearly two [AOR = 1.83 (1.23, 2.69)] times higher among mothers who chewed Khat compared to those who did not. The odds of undernutrition were three [AOR = 3.09(1.82, 5.29)] times higher among those who did not eat diversified diets and nearly four [AOR = 3.67 (1.47, 9.19)] times higher among those who live in food insecure household compared to their counterparts (Table 3).

**Table 3:** Factors associated with undernutrition among lactating mothers in chiro district, eastern Ethiopia, 2019 (*n* = 629).

Characteristic	Undernutrition		COR (95% CI)	*p*-values	AOR (95% CI)	*p*-values
	yes, *n* (%)	no, *n* (%)				
Age of mother (in years)						
15–24	39 (21.5)	142 (78.5)	1		1	
25–34	102 (28.1)	261 (71.9)	1.42 (0.93, 2.17)	0.101	0.74 (0.43, 1.26)	0.651
35–44	28 (32.9)	57 (67.1)	1.79 (1.01, 3.18)	0.047	1.04 (0.52, 2.09)	0.240
Educational status						
No formal education	137 (28.6)	342 (71.4)	1.48 (0.95, 2.29)	0.081	1.18 (0.73, 1.90)	0.408
Formal education	32 (21.3)	118 (78.7)	1		1	
Sex of Head-household						
Female	5 (12.8)	34 (87.2)	0.38 (0.15, 0.99)	0.048	0.34 (0.13, 0.94)	0.041
Male	164 (27.8)	426 (72.2)	1		1	
Wealth index						
Poor	60 (32.8)	123 (40)	1		1	
Medium	63 (31.2)	139 (68.8)	0.93 (0.61, 1.43)	0.737	0.58 (0.36, 0.95)	0.039
Rich	46 (18.9)	198 (81.1)	0.48 (0.31, 0.74)	<0.001	0.30 (0.18, 0.51)	<0.001
Family size						
≥5	130 (29.9)	305 (70.1)	1.69 (1.13, 2.54)	0.011	1.14 (0.61, 2.12)	0.831
<5	39 (20.1)	155 (79.9)	1		1	
Antenatal attendance						
No	112 (29.1)	273 (70.9)	1.35 (0.93, 1.95)	0.115	1.19 (0.76, 1.86)	0.131
Yes	57 (23.4)	187 (76.6)	1		1	
Birth interval						
First birth	9 (12.7)	62 (87.3)	1		1	
1–2 year	82 (30.2)	189 (69.8)	0.39 (0.18, 0.82)	0.015	1.755 (0.799, 3.943)	0.163
>2 years	78 (27.2)	209 (72.8)	1.16 (0.81, 1.68)	0.407	1.408 (0.626, 3.164)	0.408
Nutritional advice						
No	123 (36.8)	211 (63.2)	1.77 (1.23, 2.54)	0.002	1.63 (1.11, 2.39)	0.012
Yes	46 (15.6)	249 (84.4)	1		1	
Meal frequency per day						
<3	106 (29.8)	250 (70.2)	1.41 (0.98, 2.03)	0.061	1.16 (0.75, 1.81)	0.496
≥3	63 (23.1)	210 (76.9)	1		1	
Chewing khat use						
Yes	114 (32.9)	232 (67.1)	2.04 (1.05, 2.95)	<0.001	1.83 (1.23, 2.70)	0.002
No	55 (19.4)	228 (80.6)	1		1	
MDDW						
<5 food group	144 (30.3)	332 (69.7)	2.22 (1.39, 3.56)	<0.001	3.10 (1.82, 5.29)	<0.001
≥5 food group	25 (16.3)	128 (83.7)	1		1	
Household food security						
Insecure	163 (28.5)	408 (71.5)	3.53 (1.49, 8.39)	0.004	3.67 (1.47, 9.20)	0.002
Secure	6 (10.3)	52 (89.7)	1		1	

MDDW, minimum dietary diversity of women; COR, crude odds ratio; AOR, adjusted odds ratio.

## Discussion

The prevalence of undernutrition among lactating mothers was 26.9%. This study result is in line with the studies conducted in Bangladesh (28%) (34), Tanzania (30%) (3), Tigray (24.6%) (21), Wombera (25.6%) (29), Mi’esso (30.3%) (20). The finding of this study was relatively higher than the study conducted in Indonesia (9%) (14), Jammu and Kashmir (19.3%) (35), Kenya (22%) (36), Adama (19.5%) (27), and Nekemte (20%) (26). However, this prevalence was lower than the study done in Jordan (49.2%) (37) and Jimma (40.5%) (30). This inconsistency could be due to the difference in sample size because some of the studies were large surveys and had a relatively large sample size, as seen in the Jammu and Adama studies, while others had a smaller sample size than the present study (Kenya Study). Study area, period, and seasonal variation (since this study was done in rural residences only during summer) may also be another explanation for the observed discrepancy, which can result in food insecurity status change, while most of the previous studies had a high prevalence of food security compared to the present study. The study area (respondent residence) difference may be another possible explanation because the current study was done in a rural area. Feeding cultural practice, education level, and socioeconomic status of participants could be explanations for this observed variation.

This study reveals that household head sex was associated with undernutrition among lactating mothers. Accordingly, the odds of undernutrition among lactating mothers living in households headed by females were 66% less likely than their counterparts. The possible reason might be that a female-headed household has better autonomy to use household resources to expense for better nutritional food preparation. In addition, the female-headed household was more likely to have the self-determination to consume the recommended types of food without interference than the male-headed household (38).

Another important factor in maternal nutrition in the study area is household wealth status. It is inversely associated with maternal undernutrition. The higher the wealth status, the lower the risk of being undernourished or protected. This implies that poor households cannot meet their food consumption by buying expensive essential foods. This study revealed that the prevalence of undernutrition decreases as the economic status of respondents increased by 42% (medium) and 70% (rich) wealth status not undernourished, and this finding was supported by the study conducted in Bangladesh (34, 39).

Further, nutrition advice during pregnancy and lactation is another determinant of lactating mothers’ undernutrition. Those lactating mothers who had not gained or received nutrition advice from health workers were almost two-fold more likely to be undernourished than their counterparts. The finding also supported by the studies conducted in Adama (27) and Jimma (40). This implies that inadequate dietary intake and a lack of extra meals during these periods contribute to undernutrition. Hence, nutrition advice from health workers is imperative during this period because it increases nutrition knowledge and awareness and changes the behavior of lactating mothers as they consume adequate and quality foods, enhancing their food preferences.

In this study, substance use (chewing khat) is another important independent predictor of undernutrition and a new variable found in lactating mothers, according to our level of knowledge. Lactating mothers who used substances were almost two times more likely to be malnourished as compared to those who did not use the substances (chewing khat). This finding is consistent with the result of a study conducted on pregnant women in Gumay District, Jimma Zone (40). This implies that there is a direct relationship between substance use, khat chewing, and undernutrition. This could be due to chewing Khat causing loss of appetite and gastritis, which in turn cause low food intake and poor nutrient absorption.

Another important predictor of undernutrition was the minimum dietary diversity score of women. In this study, mothers who consume less than five food groups were three times more likely to be undernourished than those who consume more than or equal to five food groups. This implies that the consumption of a variety of food groups is protective against undernutrition. This finding is supported by a study done in the Jimma Zone (30). This could be insufficient nutrient diets, which are essential for the human body to be immune and perform metabolic activities; diversified food reflects dietary quality and improves daily nutrient and energy intake. Lactating women who do not get enough energy and nutrients in their diets risk maternal depletion and exacerbate women's undernutrition.

Household food insecurity affects the intake of an adequate quantity and quality of diet, which in turn contributes to maternal undernutrition. In the present study, lactating women from food-insecure households are nearly four times more likely to be malnourished compared to those from food-secured households. This finding is supported by a study conducted in Jimma, which revealed that women in food-insecure households have a higher risk of undernutrition than women in food-secure households (40). Another study in Bangladesh (34) also reported that food insecurity was significantly associated with lactating mothers’ undernutrition. This may be because, when food is in short supply, one coping strategy may be for women and girls to eat less, reserving more food for men and boys. These all might be leading to a lack of access to adequate, safe, and nutritious food, resulting in women's undernutrition.

Since we used a cross-sectional study design, it does not show a causality relationship between independent variables. In addition, recall bias is also one of the study's limitations due to part of the questions asked about events that occurred 24 hours and four weeks ago. These were minimized by probing the respondents about the event. Furthermore, this study was conducted among lactating mothers whose children are 6–24 months and does not generalize to those whose children are less than six months.

## Conclusions

More than one in every four lactating mothers is undernourished in rural eastern Ethiopia. This study shows male-headed households, poor wealth index, use of chewing khat, lack of nutritional feeding advice, low minimum dietary diversity, and household food insecurity were independent risk factors of undernutrition among lactating mothers. Focusing on implementing existing strategies/programs for effective nutritional interventions and poverty alleviation that enhance food security status would be essential to improving the nutritional status of lactating mothers and children. In addition, enhancing mother's awareness and access to diversified food and healthy food options through ensuring women's empowerment is essential to reducing and ending undernutrition at the community level. Furthermore, we recommend that prospective researchers explore the different qualitative reasons for the stated level of burden in rural eastern Ethiopia.

## Data Availability

The raw data supporting the conclusions of this article will be made available by the authors, without undue reservation.
